# Observation-based data-gathering method to support the assessment of the use of cultural ecosystem services in urban green spaces

**DOI:** 10.1016/j.mex.2023.102326

**Published:** 2023-08-15

**Authors:** Luís Valença Pinto, Miguel Inácio, Paulo Pereira

**Affiliations:** a Research Centre for Natural Resources, Environment and Society (CERNAS), Polytechnic Institute of Coimbra, Coimbra Agrarian Technical School, Coimbra, Portugal; b Environmental Management Laboratory, Mykolas Romeris University, Vilnius, Lithuania

**Keywords:** Mobile app, Digital data collection, Standardisation, Quantitative, ObsUGS – Mobile tool to collect observational data on the use of urban green spaces

## Abstract

Urban green spaces (UGS), such as parks, gardens, forests, and green infrastructure, supply numerous regulating, provisioning and cultural ecosystem services to urban communities, which is key for their wellbeing. To effectively plan and design UGS, it is crucial to understand how people use them and the factors that influence their usage. The diverse range of factors includes cultural, socioeconomic, demographic, seasonal, and spatial aspects, which present a challenge for studying these areas. Data collection methods based on observation enable capturing of high-quality data that reflect the behaviours in UGS, providing valuable insights for urban planners and policymakers. Standardised protocols and frameworks facilitate knowledge gathering, allowing researchers and practitioners to build upon evidence for effective urban planning and policymaking. This work aims to develop a method based on a mobile app to collect observation-based data on UGS usage efficiently.

• Mobile app to collect georeferenced information on observed activities, basic sociodemographic characteristics, time and seasonal factors, and park characteristics, including the spatial distribution of park recreational equipment.
• App optimised for fast and accurate data collection.
• Focused on cultural ecosystem services.

## Graphical abstract

**Figure:**
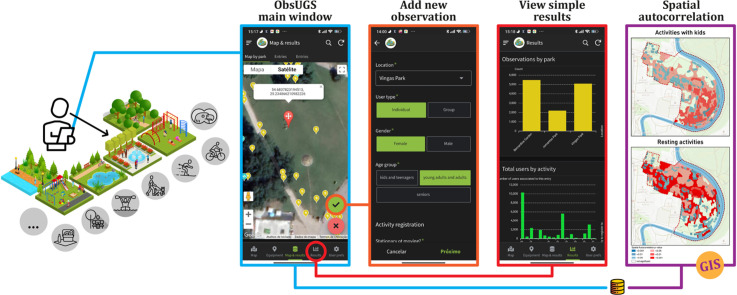

Specifications table

**Table:** 

Subject area:	Environmental Science
More specific subject area:	Cultural Ecosystem Services
Name of your method:	ObsUGS – Mobile tool to collect observational data on the use of urban green spaces
Name and reference of original method:	NA (a paper assessing the results from the first application of this method has been submitted to an Elsevier journal; under review)
Resource availability:	Mobile app creation: Appsheet.com (www.appsheet.com). Handheld digital anemometer (e.g., Benetech GM816). Handheld digital thermometer (e.g., UNI-T UT333).

## Method details

### Background

Studying the usage of urban green spaces (UGS) and their cultural ecosystem services (CES) is of utmost importance in today's urbanised world. Research in this area focuses on understanding how these green spaces are used, their benefits, and their implications for urban planning and public health. UGS, including parks, gardens, forests, and other green infrastructure, play a crucial role in promoting wellbeing [1], environmental sustainability [2], and social cohesion [3] in densely populated areas.

UGS offer numerous ecosystem services to urban communities. They provide recreation, physical activity, and relaxation opportunities, improving mental and physical health [4]. These areas contribute to carbon sequestration (e.g., [5]), mitigate the heat island effect (e.g., [6]), and filter pollutants, contributing to improved air and water quality (e.g., [7]). UGS also enhance biodiversity by providing habitats for various animal and plant species [8].

To effectively plan and design UGS, it is essential to understand how people use them and the factors that influence their usage [9]. This understanding can help inform decision-makers regarding green space location, design features, amenities, and maintenance. Data-collection methods include survey or interview questionnaires, camera and video recordings, sensors, Global Positioning System (GPS) and Geographical Information Systems (GIS) usage, and direct observations.

BALTS (Ries et al., 2009) and PA-PS [10] were innovative survey protocols for assessing park use, with both becoming popular protocols [11]. But these protocols did not collect location-specific data but rather data at the UGS level. Subsequent survey methods have combined new technologies (e.g., GPS [12] or unmanned aerial vehicles (UVA) methods [13]). Besides activity category and other characteristics, REVAMP method [14] could capture the location of activities. The Four sections quantitative questionnaire [15], is based on users’ perceptions of their park experience.

The System for Observing Play and Leisure Activity in Youth (SOPLAY) and the System for Observing Play and Recreation in Communities (SOPARC) methods were observation-based methods developed by McKenzie et al. [16,17], to obtain information on park users and their physical activities. Observations allow researchers to directly capture usage patterns, activities, and user demographics without relying on participant recall or self-reporting biases. This method can generate high-quality data that effectively reflect the actual behaviours in these spaces. However, although research on urban green spaces has grown substantially, there still needs to be more in the literature, particularly in data collection methods based on observations [11]. In contrast to subjective self-reports from face-to-face, mail, or internet surveys, observation offers a more objective means of measurement that enhances data internal validity [18]. It also allows for the simultaneous acquisition of data about the physical and social surroundings in which an activity occurs. This method has become common in Western countries for studying park use [11].

According to Joseph and Maddock [19], SOPARC was used in 85% of observation-based studies, which gave origin to other methods, e.g., the UVAs method [13]. However, SOPARC surveys have mostly relied on paper forms. A mobile app working in iOS (iPad version) was available for some time but was discontinued. The SOPARC method had some issues regarding double-counting visitors [11]. The Structured instrument [20] and EXOdES [21] are new observation-based methods. The Structured instrument focused on smaller parks and assessed individual persons rather than specific park zones, as SOPARC, thus addressing the double-count issue. EXOdES is an instrument to record park users' position and the exact time of each record entry, also allowing for collecting more detailed descriptions of users. Nevertheless, according to Chen et al. [11], most methods focused on physical activity from the perspective of health and leisure-related research, restricting their utility regarding other possible activities relevant to cultural ecosystem services (CES), such as socialisation, relaxation, or education.

One of the challenges in studying green space usage is the diverse range of factors that influence it, such as cultural, socioeconomic, demographic, seasonal, and spatial characteristics. Moreover, there is a need for standardised protocols and frameworks for conducting observational studies in UGS [22]. The lack of consistent methodologies across different studies can hinder the ability to compare findings and identify overarching trends and best practices. Standardisation would facilitate gathering knowledge in this field, allowing researchers and practitioners to build upon evidence for effective urban planning and policymaking. This work aimed to develop a method based on a mobile app to efficiently collect observation-based data on UGS usage, including data on activities performed associated with the CICES framework [23], basic sociodemographic characteristics, time and seasonal characteristics, and park characteristics comprising the spatial distribution of park recreational equipment. The main advantages of our method are related to (1) the assessment of activities based on a CES-related framework and (2) the workflow for data collection based on a mobile app optimised for a reduced time spent per observation.

### Base method – data acquisition

The method is centred on a mobile app developed on the AppSheet platform (appsheet.com). The app allows the observer to record geolocated information on observed activities (see Table 1 for a complete list of options), time-related information (season, week period, and timeslot), weather-related information (rain conditions, temperature, cloud coverage, solar exposure, wind speed) and user characteristics (age group, biological sex, individual vs group, number of users, groups with children). It also identifies and characterises recreational equipment in each UGS (e.g., benches, water fountains, waste bins, and different sports and cultural equipment types). All this was implemented in a single app, allowing for the centralisation and pre-treatment of the information, reducing posterior data preparation time. The weather variables are measured using adequate equipment, i.e., via a handheld digital anemometer and a handheld digital thermometer. We identified and recorded 33 activities based on the observed behaviour of park users, and we included an option for “Doing other activities in nature” to account for any other relevant uses not included in the established list. All data recorded in the app was geolocated over high-resolution aerial imagery to ensure spatial accuracy. The mobile app was designed to ensure consistency and efficiency in data collection. No records were saved if any data was missing. The number of users recorded depended on whether the user was an individual or part of a group. Gender options were tailored to each user type, with a “Mixed” option only available for groups. The list of available activity options in the app was restricted based on the user's motion status (in motion vs stationary), ensuring that only relevant activities were recorded. These and other rules are detailed in the section *Data Consistency and optimisation rules*.

**Table 1:** List of fields for the ‘App STRUCTURE’ sheet.

Field ID	Description
Field app name	Field designation in the spreadsheet.
Name displayed	Field name to be shown in the app (adjusted according to language).
Visible	Indication of the visibility status of the field in the app.
Dependence	Indication of the field which is dependant upon (for calculated fields).
Value	Type of value (automatic, calculated, option…)
default	Default value for the field.
NOTES	Diverse notes on the field.

Observations were recorded for individual and group users, allowing us to capture interactions with nature and differentiate between individual and shared experiences. An individual observation refers to a user engaging in an activity without interacting with other users, e.g., walking, jogging, or biking. A group observation involved two or more users sharing a common experience, such as a mother walking or playing with a child or two seniors walking together.

### Mobile application structure

**Fig. 1:**
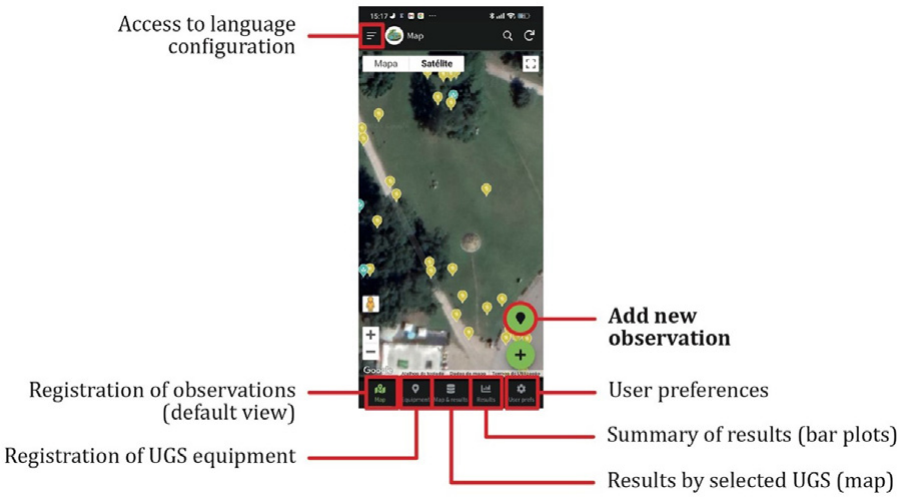
App overview, main window.

The mobile app includes a section for registering observations (main view), an equipment section, a section to do a map-based check for current data, a results section showing a resume of the data via bar plots, and a section for user preferences (Fig. 1).

The observations’ main view allows for the introduction of new observation records. To optimise data collection time, this window shows only records of the current day for the selected study area, to a maximum of 500 points. The equipment section allows for the geolocation of recreational equipment inside each study area, including, e.g. waste baskets, benches, and fountains. The map and results section allows for a global map view per selected park with all the entries. The results section shows an up-to-date set of bar plots, highlighting the results collected so far (see Figure S1a and S1b in Supplementary material for screenshot examples). Finally, the user preferences section allows for the definition of default values for specific fields used to pre-fill the form for each new observation record, reducing the time needed for data entry.

The Appsheet service allows for the creation of a mobile app using individual sheets in a Google spreadsheet format to define the structure and fields of the app. We based the app on four different sheets, each one corresponding to a different part of the app. The first is called “*App STRUCTURE*”. It contains the main structure of the app, including fields' names (currently available in three languages: English, Portuguese, and Lithuanian; see Figure S1c in Supplementary material), information on any dependence of each field, type of data origin (option, automatic, calculated), and indication of default values for specific fields (see Table 1).

The second sheet is called “*user_prefs*” and corresponds to the definition of user preferences for the app (see Table 2). The app sheet service includes authentication services, which, by default, use Google accounts to verify app users. The user preference section keeps the default value preferences associated with each user to support faster fieldwork. The third table is called “*equipment*”, corresponding to the information collected for each recreational equipment found in each park, or UGS (see Table 3). The last sheet (“*main data*") corresponds to the fields associated with each observation (Table 4).

**Table 2:** List of fields for the ‘user_prefs’ sheet.

Field name	Description
user_ID	System unique ID.
user-email	system user email.
group	Option regarding the default value for the type of user.
gender	Option regarding the default value for gender.
kids	Option regarding the default value for groups with kids.
age-group	Option regarding the default value for the age group.
park	Option regarding the default value for the UGS in analysis.
motion	Option regarding the default value for the motion status.
es-class	Option regarding the default value for ES activity.
wea-clouds	Option regarding the default value for clouds (weather).
wea-rain	Option regarding the default value for rain (weather).
wea-snow	Option regarding the default value for snow (weather).
wea-wind	Option regarding the default value for windspeed (weather).
temp	Option regarding the default value for temperature (weather).
exposure	Option regarding the default value for solar exposure.

**Table 3:** List of fields for the ‘equipment’ sheet.

Field name	Description
unique-ID	system unique ID.
user	system user email.
coordinates	coordinates in decimal degrees, WGS84 system.
latitude	Latitude value in decimal degrees, based on 'coordinates'.
longitude	Longitude value in decimal degrees, based on 'coordinates'.
date	Date of the record.
time	Time of the record.
park	Identification of the corresponding UGS.
type	Equipment type (see Table 5).
orientation	Bench orientation regarding closest path (benches only).
distance to path	Distance to the closest path (benches only)
degree of tree cover	Degree of tree coverage (benches only) (see Table 5).
viewshed	Type of viewshed from the equipment (benches only) (see Table 5).
visual exposure	Level of visual exposure of the equipment (benches only) (see Table 5).
photo	Link to photo of the equipment.

**Table 4:** List of fields for the ‘main data’ sheet, used for the observations.

Field ID	Field name	Name displayed	Visible	Method	NOTES
1	unique-ID	–	no	Automatic	Unique ID attributed by the system.
2	user	–	no	Automatic	The email of the user adding the entry.
3	coordinates	–	no	Automatic	coordinates in decimal degrees, WGS84 system (‘lat, long' format), associated with the point identified by the observer in the map.
4	latitude	–	no	Calculated	Latitude value in decimal degrees, based on field 3.
5	longitude	–	no	Calculated	Longitude value in decimal degrees, based on field 3.
6	date	–	no	Automatic	Current data of the observation, format ‘dd.mm.yyyy’. System value
7	time	–	no	Automatic	Current observation time, format 'hh:mm:ss’.
8	timeslot	–	no	Calculated	Calculated based on the current time. Morning: 08h30–12h30, Afternoon: 12h30–17h00, and Evening: 17h00–21h30.
9	weekday	–	no	Calculated	Weekday according to the system. 1 – Sunday, 2 – Monday, 3 – Tuesday, 4. Wednesday, 5 – Thursday, 6 – Friday, 7 - Saturday
10	weekday-ID	–	no	Calculated	Weekday according to the system (1 to 7).
11	week-period	–	no	Calculated	The internal calculation, based on the weekday system value (Workdays vs. weekends).
12	week-period-ID	–	no	Calculated	1 for workdays, 2 for weekends.
13	location	Location	yes	Option	Study site name from the list added to the app (see Table 5).
14	user-type	User type	yes	Option	Single user vs group (see Table 5).
15	user-type-ID		no	Calculated	ID of the selected option (see Table 5).
16	gender	Gender	yes	Option	Male, female, or other (see Table 5).
17	gender-ID	gender ID	no	Calculated	ID of the selected option (see Table 5).
18	user-type-kids	Adult(s) with kids?	depends	Option	True or False.
19	user-type-kids-ID	user-type-family-ID	no	Calculated	ID of the selected option (see Table 5).
20	age-group	Age group	yes	Option	kids and teenagers young adults and adults seniors mixed (see Table 5).
22	user-number	Number of users associated with this entry	depends	Numerical input	User input.
23	motion	Stationary or moving?		Option	Is the observed user in motion or stationery?
40	exposure	Sun exposure	yes	Option	Is the user under direct solar exposure? (only for stationary users; see Table 5).
41	exposure-ID	exposure ID		Calculated	ID of the selected option (see Table 5).
24	title-ES	Activity registration	yes	–	Section separator / title.
27	es-class	The activity to register is:	yes	Option	Observed activity selected from options (see Table 5).
28	es-class -ID	ES class ID	no	Calculated	ID of the selected option (see Table 5).
29	es-class- other	Other recreational use	yes	Option	Select / add another activity not listed in the main list of C.ES
30	es-class- other-ID	ES class sub other ID	no	Calculated	ID of the selected option (see Table 5).
31	title-val-weather	Weather info	yes	–	Section separator / title.
32	weather-clouds	Sky conditions	yes	Option	Cloud coverage (see Table 5).
33	weather-clouds-ID	weather cloud ID	no	Calculated	ID of the selected option (see Table 5).
34	weather-rain	Rain	yes	Option	Rain conditions (see Table 5).
35	weather-rain-ID	weather rain ID		Calculated	ID of the selected option (see Table 5).
36	weather-wind	Wind speed	yes	Option	Wind speed in m/s.
37	weather-ground-snow	Ground covered by snow?	yes	Option	Yes or no?
38	weather-ground-snow-ID	Ground snow coverage ID		Numerical input	ID of the selected option (see Table 5).
39	temperature	Local temperature	yes		Air temperature in °C.

### Keywords/definitions

*Observer* – the app user responsible for the collection of accurate observation data.

*Single user observation* – corresponds to an observation of a single user performing an activity without other users.

*Group observation* – corresponds to two or more users sharing a common experience (e.g., a mother and a child or two seniors walking along). Registered as a single observation, one activity but with multiple users.

### Options for observation and equipment fields

The survey tool is based on closed questions with concrete and limited options. Except for temperature and wind speed, each field regarding observations and equipment has a pre-set list of data harmonisation and comparability options. Table 5 shows each field's options for observations and available recreational equipment.

**Table 5:** Field options.

Options for observations *						Options for equipment **		
ES options		Language options		Clouds options (weather)		Equipment type options		
**id**	**Option**	**id**	**Option**	**id**	**Option**	**id**		**Option**
31,401	Walking the dog	1	English	1	clear sky	1		Bench
31,402	Walking, strolling, hiking	2	Português	2	partially overcast	2		Water fountain
31,403	Jogging, running	3	Lithuanian	3	overcast	3		Garbage bin
31,501	Doing winter sports					4		Toilets
31,940	Fitness	**UGS options**		**Rain options (weather)**		51		Sports (fitness)
31,930	Group sports	**id**	**Option**	**id**	**Option**	52		Sports (basket)
31,301	Biking	1	Vingis Park	1	no rain	53		Sports (tennis)
31,310	Skating	2	Bernardino Garden	2	light rain / showers	54		Sports (volleyball)
31,201	Boating, canoeing	3	Jomantas Park	3	heavy rain	55		Sports (badminton)
31,202	Swimming, snorkelling, diving			4	light snow	56		Sports (Frisbee)
31,101	Fishing	**Motion options**		6	heavy snow	61		Culture (general eq.)
31,950	Activities with kids	**id**	**Option**	5	thunderstorm	62		Culture (info panel)
31,951	Winter activities with kids	1	stationary			63		Culture (bird feeder)
31,920	Reading	2	moving	**Snow options (weather)**		64		Culture (other)
23,502	Resting, relaxing			**id**	**Option**	7		Playground
31,602	Meeting people	**User type options**		1	No	8		Bike parking
31,601	Barbequing, picnicking	**id**	**Option**	2	Yes	42		Dog trash bags
32,199	Yoga / meditation	1	Individual			9		Picnic area
32,000	Experiential and aesthetic activities	2	Group	**Solar exposure options**				
31,910	Doing other activities in nature			**id**	**Option**	**Equipment orientation**		
		**Gender options**		1	in the shade	**id**		**Option**
**ES options ('other')**		**id**	**Option**	2	transition area	1		Facing path
**id**	**Option**	1	Female	3	exposed	2		Not facing path
4	Photography / videography	2	Male					
5	Local administration activities	3	Mix (group)			**Vegetation cover**		
6	Proselytising					**id**		**Option**
7	Playing chess or other board game	**Age group options**				1		None
9	Sunbathing	**id**	**Option**			2		Partial
10	Feeding fauna	1	kids and teenagers			3		Dense
11	Activities with kids in water	2	young adults & adults					
12	Other water activities	3	seniors			**Viewshed dimension**		
13	Working with computer	4	mixed			**id**		**Option**
14	Education					1		Narrow / short
15	Collecting plants					2		Mid-sized
19	Collecting timber					3		Wide / long
16	Watching animals							
17	Playing with the dog					**Visual exposure**		
18	Roler skating					**id**		**Option**
						1		High
						2		Average
						3		Low

* Options for each field in the 'Main data' table corresponding to the observation records.

** Options for each field in the 'equipment' table related to the record of recreational equipment.

### Data consistency and optimisation rules

To ensure data consistency and to optimise collection time, a set of rules were developed and applied in the app to prevent errors in each observation record. These rules are shown in Table 6. Figures S1d, S1e, and S1f in Supplementary Materials show examples of how the form is affected by these rules.

**Table 6:** Data consistency and optimisation rules.

Base field	Affected field	Charact.	Rule
[user-type]	[gender]	Options	IF [user-type] = ‘*single user*’ THEN exclude option ‘*Mix (group)*’ for [gender]
[user-type]	[age-group]	Options	IF [user-type] = ‘*single user*’ THEN exclude option ‘*mixed*’ for [age-group]
[user-type]	[user-type-kids]	Visibility + value	IF [user-type] = ‘*single user*’ THEN hide [user-type-kids] field AND [user-type-kids] = 0
[user-type]	[user-number]	Visibility + value	IF [user-type] = ‘*single user*’ THEN hide [user-number] field AND [user-number] = 1
[motion]	[es-class]	Options	If [motion] = ‘*stationary*' THEN show a list of motion-related activities ELSE show a list of stationary-related activities
[motion]	[exposure]	Visibility	If [motion] = ‘*moving*’ THEN hide [exposure] AND [exposure] = ‘’
[es-class]	[es-class- other]	Visibility	If [es-class] = ‘*Doing other activities in nature*’ THEN show [es-class-other] field

### Appsheet formulas for data consistency and optimisation

Appshet formulas were used in different fields in the main form to optimise the registration of new observations, implementing the rules identified in the previous section. Specific formulas were used to (1) add automatic values for automated fields (e.g., unique-ID, user, coordinates, date, time, timeslot, weekday, week period) so that the app user does not waste time recording this information by hand; (2) add pre-set values for specific fields, which are based on the value of other variables influencing answer options (e.g., field ‘es-class’); and (3) add pre-set values for specific fields, based on default values defined by the app user in the User preference section (e.g., fields ‘location’ or ‘user-type’). Field formulas can be seen in Table S1 in supplementary materials.

### Fieldwork guidelines

**Fig. 2:**
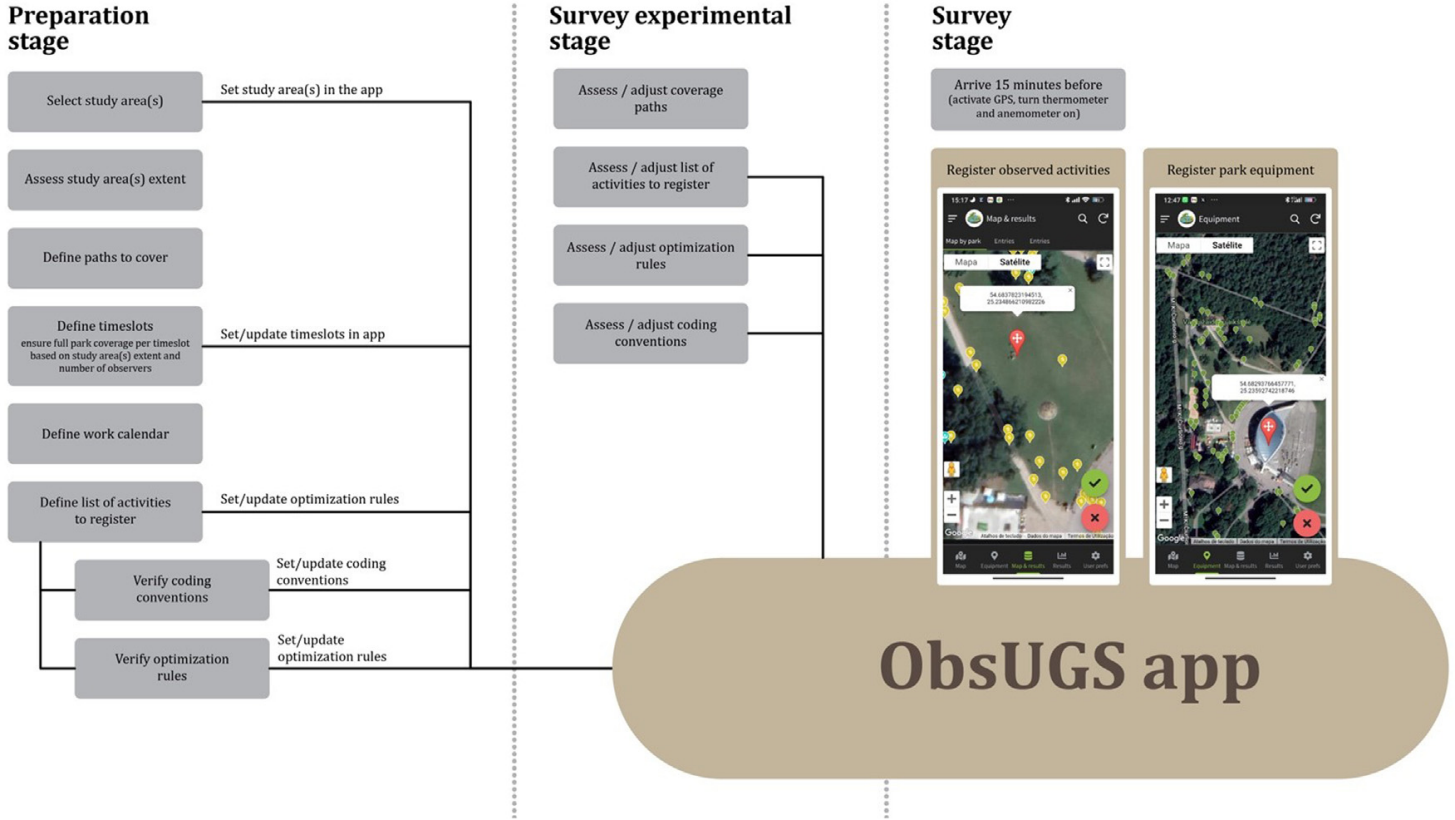
Flowchart with necessary steps for the different stages of the method.

A set of specific steps should be followed to ensure adequate results. These steps cover three different stages: the preparation stage, the survey experimental stage, and the survey stage (Fig. 2).

### Spatial coverage

The study area must be covered by walking along the available paths. Each study area must be fully covered at least once per timeslot. If the study area is small, the observer can walk multiple times along the different paths to ensure even coverage of the parking area. A previous analysis should be made to ensure that all relevant paths are included in the study. Figure S2 in Supplementary Materials shows an example of identifying walking paths to be covered for a study area in Vilnius, Lithuania.

### Work calendar

A minimum period of one week for fieldwork should be considered per UGS under analysis. This period includes workdays and weekends, ensuring a 7-day coverage per UGS. If the number of UGS to cover exceeds 1, a work calendar should be prepared to ensure efficient and adequate coverage of all the study areas.

### Time-related guidelines

For seasonal analysis, the same amount of time should be given for the assessed seasons, covering at least the summer and the Winter seasons. Different timeslots can be defined and assessed. In our study case in Vilnius, Lithuania (publication under development), three timeslots were defined and assessed due to park size and the time necessary to cover each park in each timeslot fully. The timeslots defined were *Morning* (08h30–12h30), *Afternoon* (12h30–17h00), and *Evening* (17h00–21h30). Depending on safety concerns and the geographical location of the study area, evening timeslots may be excluded during winter. Based on our experience, the minimum time spent in each timeslot per park should be 3.5 h. As stated in the previous section, each park should be covered seven days a week to assess workdays and weekend usage. This frequency of coverage (days and time coverage per timeslot) allowed us to obtain reliable estimates of park user characteristics and activity engagement.

### Multiple observers

A single observer can develop the fieldwork, but two observers can work together. When working with two observers, they should collect data together, walking along the designated paths within the study sites. When encountering areas with a high concentration of users (such as open spaces, playgrounds, or fountain areas), the observers should communicate between them regarding which areas or users they are covering to avoid repetitions. The observers should spend at least 10 min in these crowded areas to ensure comprehensive coverage.

### Survey experimental phase

Before starting data collection, at least 2 days should be used for testing the app in the field. This time should be used to assess and adjust (i) coverage paths, (ii) the list of ecosystem services-related activities, (iii) the optimisation rules, and (iv) coding conventions (see below).

### Observation preparation

Before going to the study areas, prepare all the equipment, check battery levels, and pack a power bank for extra charges. Arrive at the site at least 15 min before the official start of data collection. Turn on all devices that might need a starting time to adjust to local conditions, e.g., activate the GPS of the mobile device and let the thermometer adjust to external conditions.

### Specific coding conventions

Each user or group is recorded only once per timeslot unless they are observed engaging in a different activity. For playground areas, where parents are often at a distance from their children, time is needed to observe the persons in the area to assess groups and group characteristics, such as size or gender. It is necessary to dedicate at least 5 min to assess these issues. Specific conventions were defined to ensure data consistency regarding registering specific activities (Table 7). According to the characteristics of the study area and its users, extra-specific coding conventions might be defined. This is assessed in the experimental phase of the survey.

**Table 7:** Rules for the classification of specific activities.

Situation	Rule
Users in activities with children or baby strollers	Classify as “Activities with kids”
Users walking dogs (with no children)	Classify as “Walking the dog”
Two or more users walking and interacting between them (with no children)	Classify as “Meeting people”
Two or more users walking together but not interacting (with no children)	Classify as “Walking”
Users with children	Gender information is considered only for adults
Group exclusively made of children	Consider gender of children

### Data analysis – application suggestions

The method allows for data collection, which can be used to answer diverse research questions, e.g.: (i) which main user characteristics influence park usage? (ii) are there any differences in park activities according to time-related variables, such as the season, week period, and time of day?; (iii) Are there any variations in user characteristics per park according to the season, week period, and time of day?; (iv) Is there an influence of weather variables in park activities?; (v) Are there differences in the spatial distribution of activities inside each park?; (vi) Which factors influence the use of UGS?; (vii) are there any spatial relations between preferred activities and the spatial distribution of recreational equipment?

For analysis purposes regarding weather variables, the windspeed values can be converted into classes of the Beaufort wind scale [24] (Table 8), and the external temperature can be converted to temperature comfort zones according to a scale adapted from [25] and ASHRAE 55 Parameters [26] (Table 9).

**Table 8:** Beaufort wind scale (based on [13]).

Wind speed	Beaufort class
0.28 m/s	Calm
1.39 m/s	Light Air
3.06 m/s	Light Breeze
5.28 m/s	Gentle Breeze
7.78 m/s	Moderate Breeze
10.56 m/s	Fresh Breeze
13.61 m/s	Strong Breeze
16.94 m/s	Near Gale

**Table 9:** Temperature comfort (based on [14] and [15]).

Temp.	Comfort zone
0 °C	Sub-zero (negative)
16 °C	Uncomfortable (too cold)
22 °C	Slightly uncomfortable (cold)
29 °C	Comfortable
37 °C	Slightly uncomfortable (hot)
45 °C	Uncomfortable (too hot)

The data structure can be directly used in correlation and / or factor analyses. The observed activities can be grouped into categories for analysis for a more straightforward assessment. In our case study (publication under development), the observed activities (33) were divided into 8 groups: *Activities with children, Social activities, Walking, Sports & water activities, Activities with dogs, Resting activities, Experiential & aesthetic activities*, and *Other activities*.

A Kernel density estimation analysis can be applied to assess spatial heat maps of observations in each UGS under analysis. Spatial autocorrelations between groups of stationary activities and the available recreational equipment can be assessed through a spatial autocorrelation (Global Moran's I) analysis [27] based on both feature spatial locations and feature values (e.g., locations of users engaging in activities with children vs. playground locations).

### Case study application – resume from publication under revision

The method was applied in a study developed in Vilnius, Lithuania. A total of 3 UGS were assessed (Vingis Park, Jomantas Park, and Bernardino Garden). The study sites were assessed in both summer (July 2021) and winter (January and February 2022) seasons. Each park was assessed one full week per season, Monday to Sunday, for three different timeslots (morning – 8h00–12h30, afternoon – 12h30–17h00, evening – 17h00–21h30).

**Fig. 3:**
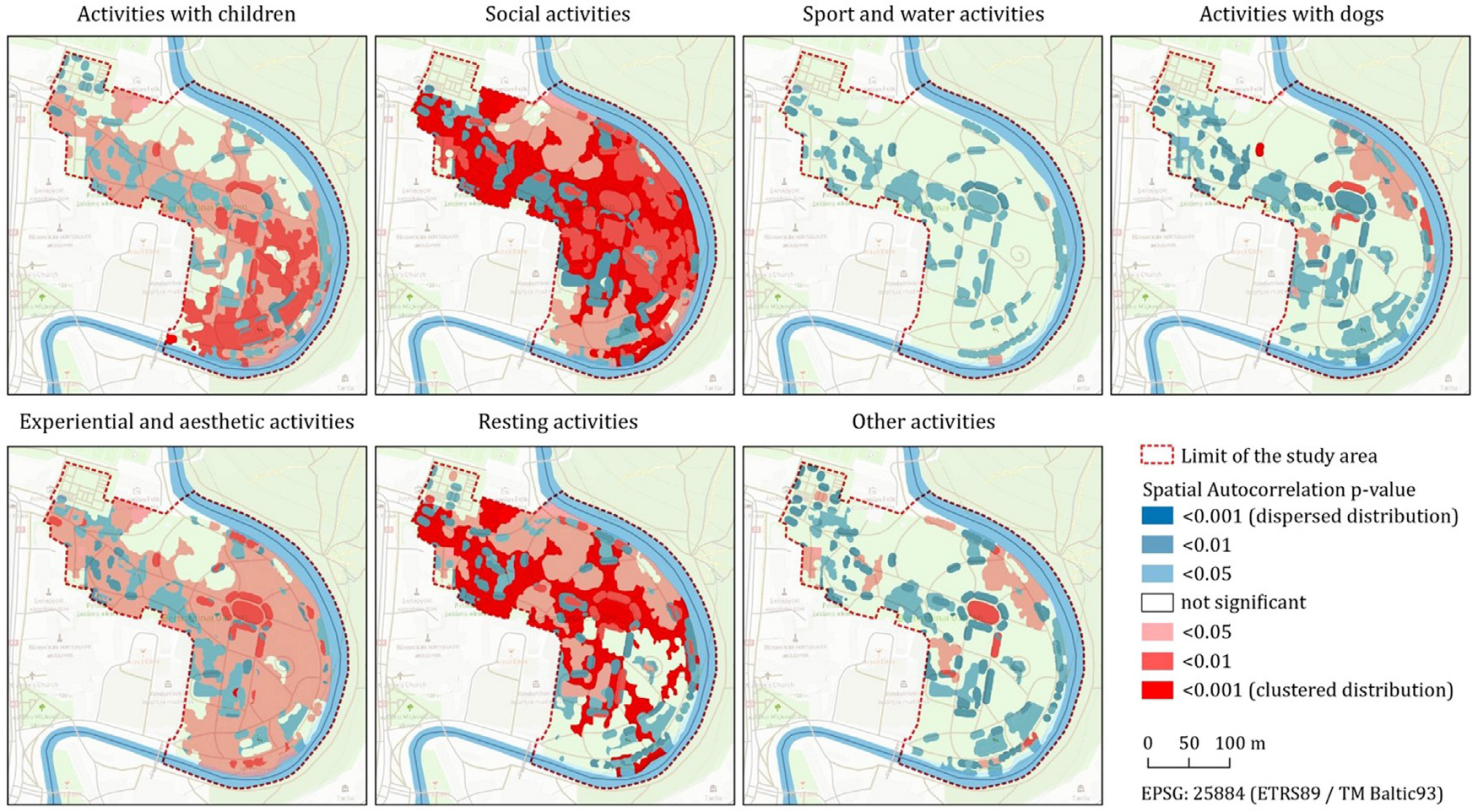
Example of results from a spatial autocorrelation analysis between ES-related activity groups and land use. Red indicates higher positive values, showing a clustered distribution of points. Blue represents lower negative values, showing a dispersed distribution of points (*n* = 2969).

19,992 observations were recorded (70.4% during summer), corresponding to 40,317 users, covering both stationary and moving users. Significant differences (*p*<0.05) were observed at seasonal, week periods, and time of day periods regarding variations in activities per park and user characteristics per park. Significant differences were also assessed regarding activities related to weather variables. Variations were also studied related to the spatial distribution of activities in the study areas. Parks with higher diversity of equipment (sports and cultural) showed a high seasonal difference in the number of activities. The number of users was high in the summer for some activities (e.g., Activities with kids, Social, Sports and water activities). Jomantas Park showed low variability in user characteristics compared to the other two parks. Precipitation, wind speed, and air temperature influenced users' activities. The spatial distribution of activities mainly depended on the available equipment rather than the park's size. The distribution of stationary activities showed spatial correlation with park characteristics (e.g., distribution of urban infrastructure such as benches, playgrounds, sport and fitness equipment, or proximity to water features). Fig. 3 shows an example of the results from the spatial autocorrelation analysis for the Bernardino Garden study area [27]. These specific results show a high spatial autocorrelation with observed activities with kids and the location of two playgrounds and surrounding lawns in the southeastern area of the park while resting activities avoid those same areas.

The study findings demonstrate that leisure activities in UGS are influenced by season, weather, and timing. Climate change is expected to bring about permanent and significant changes in UGS usage [28]. This aligns with previous research indicating climate change impacts UGS utilisation [29]. Consequently, a progressive shift from winter to summer usage patterns is anticipated due to increased usage pressure.

The demographic composition of UGS users is another vital consideration. Senior citizens were found to be prevalent users, particularly in Jomantas Park and during morning and evening periods in other parks. With the projected population ageing, it is expected that the elderly will spend more time in various types of UGS [30]. This corresponds to seniors' growing need for social interaction and contact with nature, as identified in previous studies [31]. However, this trend, coupled with high park user density, particularly in Bernardino Garden, may compromise the quality of user experiences due to overcrowding.

Analysis of UGS visitation time slots revealed that although fewer users were observed during winter overall, more users were registered in the morning and afternoon periods compared to summer season in some parks. This underscores the importance of considering visitation patterns in urban planning and necessitates efficient management of UGS resources, especially amidst increasing urban densification.

Considering these factors, it is crucial to design UGS to meet the needs of present and future users while considering expected climate conditions. The World Health Organization recommends treating UGS projects as long-term social and public health investments, requiring adequate planning and maintenance [32]. Consequently, the design process should be adaptable, allowing for functional adjustments to accommodate changes in climate, demographics, and urban densification. These measures are essential to recognise UGS as valuable resources that contribute to the wellbeing of individuals and communities, ensuring their sustained benefits amidst ongoing changes.

Based on the results, amongst other measures, an overall assessment of the current UGS is needed, looking at UGS's role in the context of climate change. There is an urgent need to identify issues related to water shortage, species growth and adaptation, and infrastructure existence. The reduction of the urban heat-island effect is essential. A shift is needed towards species adapted to the foreseeable dryer and hotter climates, considering the life expectancy of plants in new and replanting projects.

Results revealed novel insights concerning differences in group size between seasons, with the winter period showing higher numbers of users in specific timeslots for two of the three parks. These results highlight the complexity of UGS usage and the urgency to do more research focusing on multi-dimensional and multi-temporal analysis regarding UGS usage. They also highlight the need to plan UGS for climate change scenarios, considering a probable increase in usage pressure with warmer winters.

## Limitations and possible developments

The main limitation of the method is related to its main characteristic: an observation-based method. Intense fieldwork is needed to ensure the maximum amount of data to be collected. This is particularly relevant in areas of intense use. To answer this limitation, the mobile tool was developed focused on speeding the data collection process (e.g., using choice buttons instead of drop-down menu options for variables with few options) while giving consistency to the collected data (i.e., reducing inaccurate data collection through the application of specific rules that prevent incorrect / not relevant options to be available for selection). The app has a multiple-language implementation covering English, Lithuanian, and Portuguese. Further languages can be added. The definition of study areas and the list of ES activities are 'hard' coded into the base spreadsheet. A user-friendly operation can be achieved by adding an “App definitions” section. This section will allow the users to easily manage the definition / changes in UGS areas, ES activities, and other options that might be useful to change or update.

## Conclusions

Studying the usage of UGS is crucial for understanding their role in promoting wellbeing, environmental sustainability, and social cohesion. However, more literature is needed, particularly concerning data collection methods based on observations. Addressing these gaps by prioritising and promoting observation-based research can yield valuable insights for optimising UGS's design, management, and utilisation. Furthermore, data collection should be framed under the CICES framework for added usability. The exposed method allows for efficient and fast collection of data regarding the usage of CES in UGS, identifying the most relevant CES, and analysing the relations between sociodemographic, weather, seasonal, and spatial characteristics of the UGS.

## Ethics statements

No private data is collected that can identify UGS users in any way. All the data collected is used solely to evaluate nature areas based on user preferences. No images were collected except for the collection of information regarding recreational equipment inside the UGS.

## CRediT authorship contribution statement

**Luís Valença Pinto:** Conceptualization, Methodology, Software, Data curation, Writing – original draft. **Miguel Inácio:** Writing – review & editing. **Paulo Pereira:** Supervision, Conceptualization, Methodology, Writing – review & editing.

## Declaration of Competing Interest

The authors declare that they have no known competing financial interests or personal relationships that could have appeared to influence the work reported in this paper.

## Data Availability

Data will be made available on request.

## Appendix Supplementary materials

Image, application 1
