# Supplementary material for: Observation-based data-gathering method to support the assessment of the use of cultural ecosystem services in urban green spaces
Source: MethodsX. 2023 Aug 15;11:102326. doi: 10.1016/j.mex.2023.102326 (PMC10462849; doi:10.1016/j.mex.2023.102326)
Supplement: Supplementary file 1 [file mmc1.docx]

**Supplementary material** for Valença Pinto et al. 2023. Observation-based data-gathering method to support the assessment of the use of cultural ecosystem services in urban green spaces. MethodsX.

Table S1 – Field formulas in Appsheet

| **Field ID** | **Field name** | **Appsheet formulas** | **Notes** |
| --- | --- | --- | --- |
| 1 | unique-ID | =UNIQUEID() | Creates a unique identifier (alphanumeric) for each entry. |
| 2 | user | =USEREMAIL() | Associate the user’s email to the observation |
| 3 | coordinates | =HERE() | Sets coordinates as geographical coordinates in WGS84 ‘lat,long’ format. |
| 4 | latitude | =LAT([coordinates]) | Saves latitude coordinate separately, from field ‘coordinates’. |
| 5 | longitude | =LONG([coordinates]) | Saves longitude coordinate separately, from field ‘coordinates’. |
| 6 | date | TODAY() | Sets the date as the current date at the moment of creating a new entry. |
| 7 | time | TIMENOW() | Sets the time as the current time at the moment of creating a new entry. |
| 8 | timeslot | ifs(  TIMENOW()<=TIME(08:00:00), "Night",  TIMENOW()<=TIME(12:30:00), "Morning",  TIMENOW()<=TIME(17:00:00), "Afternoon",  TIMENOW()<=TIME(21:30:00), "Evening",  TIMENOW()<=TIME(23:59:99), "Night"  ) | Sets the timeslot according to the defined time intervals. |
| 9 | weekday | SWITCH(  WEEKDAY(NOW()),  1, "Sunday",  2, "Monday",  3, "Tuesday",  4, "Wednesday",  5, "Thursday",  6, "Friday",  "Saturday",  ) | Sets the weekday according to [weekday-ID], as identified by the Appsheet system. |
| 10 | weekday-ID | WEEKDAY(NOW()) | Sets the number of the day of the week. |
| 11 | week-period | SWITCH(  WEEKDAY(NOW()),  1, "weekend",  2, "workdays",  3, "workdays",  4, "workdays",  5, "workdays",  6, "workdays",  "weekend",  ) | Sets the week period, according to the ‘weekend’ and ‘workdays’ division. Based on the field [week-period-ID]. |
| 12 | week-period-ID | SWITCH(  WEEKDAY(NOW()),  1, 2,  2, 1,  3, 1,  4, 1,  5, 1,  6, 1,  2,  ) | Sets the period of the week in a numerical format, according to field [date]. |
| 13 | location | LOOKUP(USEREMAIL(), "slc_user_prefs", "user-email", "park") | Pre-sets the location (park) under analysis, as defined by the app user in the ‘User preferences’ section. |
| 14 | user-type | LOOKUP(USEREMAIL(), "slc_user_prefs", "user-email", "group") | Pre-sets the user type, as defined by the app user in the ‘User preferences’ section. |
| 15 | user-type-ID | switch(  [Language Option],  "Português", LOOKUP([user-type], "OP_user_type", "PT", "ID"),  LOOKUP([user-type], "OP_user_type", "EN", "ID"),  ) | Sets the user type ID based on the field [user-type] and according to the language choice set up by the app user. |
| 16 | gender | LOOKUP(USEREMAIL(), "slc_user_prefs", "user-email", "gender") | Pre-sets the gender type, as defined by the app user in the ‘User preferences’ section. |
| 17 | gender-ID | switch(  [Language Option],  "Português", LOOKUP([gender], "OP_gender", "PT", "ID"),  LOOKUP([gender], "OP_gender", "EN", "ID"),  ) | Sets the gender-ID based on the field [gender] and according to the language choice set up by the app user. |
| 18 | user-type-kids | LOOKUP(USEREMAIL(), "slc_user_prefs", "user-email", "kids") | Pre-sets the information on activities with children, as defined by the app user in the ‘User preferences’ section. |
| 19 | user-type-kids-ID | switch(  [user-type-kids],  "FALSE", "",  "1",  ) | Sets the user-type-kids-ID based on the field [user-type-kids] and according to the language choice set up by the app user. |
| 20 | age-group | LOOKUP(USEREMAIL(), "slc_user_prefs", "user-email", "age-group") | Pre-sets the age group as defined by the app user in the ‘User preferences’ section. |
| 22 | user-number | ifs(  [user-type-ID]=1, "1",  AND([user-type-kids]=TRUE,[gender-ID]=3), "3",  AND([user-type-ID]=2,[gender-ID]<>3), "2",  AND([user-type-ID]=2,[user-type-kids-ID]=0), "2") | Pre-sets the user number associated to the record according to user type, presence of children, and gender (check table 7 for rules). |
| 23 | motion | LOOKUP(USEREMAIL(), "slc_user_prefs", "user-email", "motion") | Pre-sets the motion status as defined by the app user in the ‘User preferences’ section. |
| 40 | exposure | if(([motion]<>"stationary"),"",LOOKUP(USEREMAIL(), "slc_user_prefs", "user-email", "exposure")) | Pre-sets the solar exposure information based on the [motion] status, and based on any pre-set values defined by the app user. . |
| 41 | exposure-ID | switch(  [Language Option],  "Português", LOOKUP([exposure], "OP_exposure", "PT", "ID"),  LOOKUP([exposure], "OP_exposure", "EN", "ID"),  ) | Sets [exposure-ID] value based on the field [exposure] and according to the language choice set up by the app user. |
| 24 | title-ES | - | USED ONLY FOR CREATING HEADERS IN THE FORM |
| 27 | es-class | ifs(  AND([motion]="stationary",[user-type-ID]=1),"Resting, relaxing",  AND([motion]="moving",[user-type-ID]=1),"Walking, strolling, hiking",  [user-type-kids-ID]=1,"Activities with kids",  AND([motion]="stationary",[user-type-kids-ID]=0,[user-type-ID]=2,[age-group]="seniors"),"Resting, relaxing",  AND([user-type-family-ID]=0,[user-type-ID]=2,[age-group]<>"seniors"),"Meeting people",  AND([motion]="moving",[user-type-kids-ID]=0,[user-type-ID]=2,[age-group]="seniors"),"Walking, strolling, hiking"  ) | Pre-sets the activity associated to the record according to motion status, age group, and presence of children (check table 7 for rules). |
| 28 | es-class -ID | switch(  [Language Option],  "Português", LOOKUP([es-class], "OP_ES", "PT", "ID"),  LOOKUP([es-class], "OP_ES", "EN", "ID"),  ) | Sets the es-class-ID based on the field [es-class] and according to the language choice set up by the app user. |
| 29 | es-class- other | - | No pre-set |
| 30 | es-class- other-ID | switch(  [Language Option],  "Português", LOOKUP([es-class-other], "OP_ES_other", "PT", "ID"),  LOOKUP([es-class-other], "OP_ES_other", "EN", "ID"),  ) | Sets the es-class-other-ID based on the field [es-class-other] and according to the language choice set up by the app user. |
| 31 | title-val-weather | - | USED ONLY FOR CREATING HEADERS IN THE FORM |
| 32 | weather-clouds | LOOKUP(USEREMAIL(), "slc_user_prefs", "user-email", "wea-clouds") | Pre-sets the clouds information with the default value defined by the app user in the ‘User preferences’ section. |
| 33 | weather-clouds-ID | switch(  [Language Option],  "Português", LOOKUP([weahter-clouds], "OP_weather_clouds", "PT", "ID"),  LOOKUP([weahter-clouds], "OP_weather_clouds", "EN", "ID"),  ) | Sets the weather-clouds-ID based on the field [weather-clouds] and according to the language choice set up by the app user. |
| 34 | weather-rain | LOOKUP(USEREMAIL(), "slc_user_prefs", "user-email", "wea-rain") | Pre-sets rain status with the default value defined by the app user in the ‘User preferences’ section. |
| 35 | weather-rain-ID | switch(  [Language Option],  "Português", LOOKUP([weahter-clouds], "OP_weather_clouds", "PT", "ID"),  LOOKUP([weahter-clouds], "OP_weather_clouds", "EN", "ID"),  ) | Sets [weather-rain-ID] based on the field [weather-rain] and according to the language choice set up by the app user. |
| 36 | weather-wind | LOOKUP(USEREMAIL(), "slc_user_prefs", "user-email", "wea-wind") | Pre-sets the wind speed with the default value defined by the app user in the ‘User preferences’ section. |
| 37 | weather-ground-snow | LOOKUP(USEREMAIL(), "slc_user_prefs", "user-email", "wea-snow") | Pre-sets the information of snow in the ground with the default value defined by the app user in the ‘User preferences’ section. |
| 38 | weather-ground-snow-ID | switch(  [Language Option],  "Português", LOOKUP([weather-ground-snow], "OP_weather_snow_ground", "PT", "ID"),  LOOKUP([weather-ground-snow], "OP_weather_snow_ground", "EN", "ID"),  ) | Sets [weather-ground-snow-ID] based on the field [weather-ground-snow] and according to the language choice set up by the app user. |
| 39 | temperature | LOOKUP(USEREMAIL(), "slc_user_prefs", "user-email", "temp") | Pre-sets the information of air temperature with the default value defined by the app user in the ‘User preferences’ section. |
| Names in capitals with closed brackets correspond to Appsheet functions, e.g., UNIQUEID() provides a unique identifier for each new entry.  Square brackets indicate a field name, e.g., [coordinates] | | | |

| 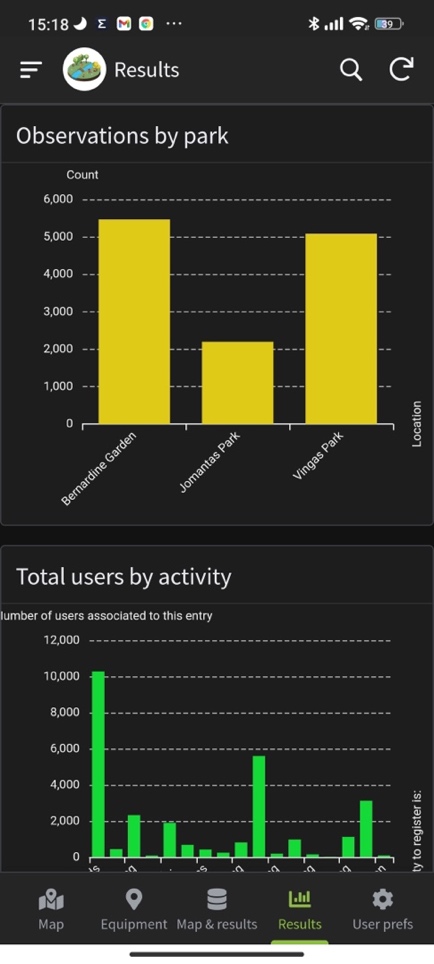 | 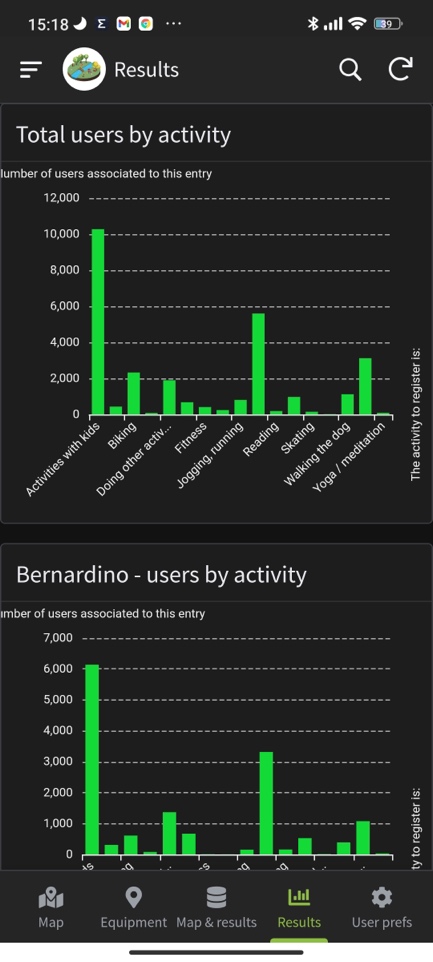 | 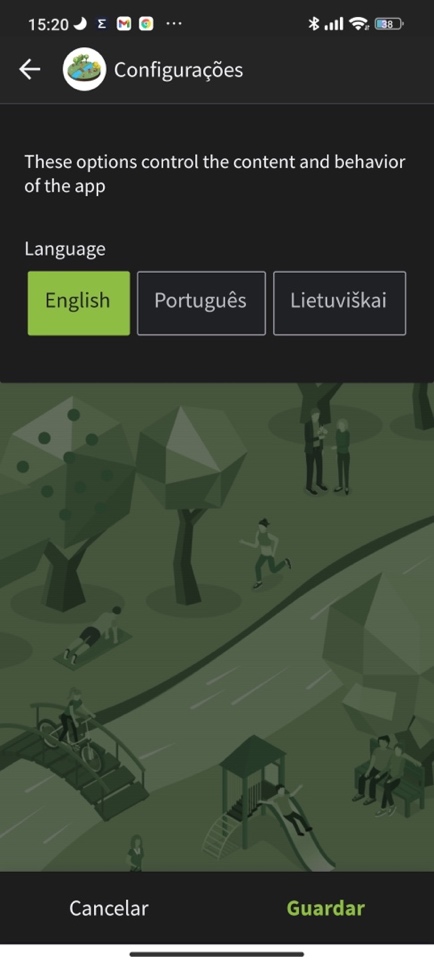 |
| --- | --- | --- |
| a | b | c |
| 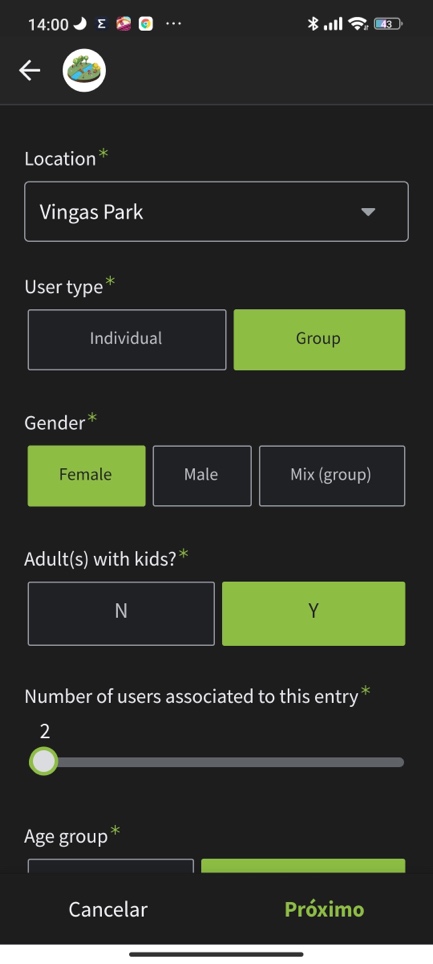 | 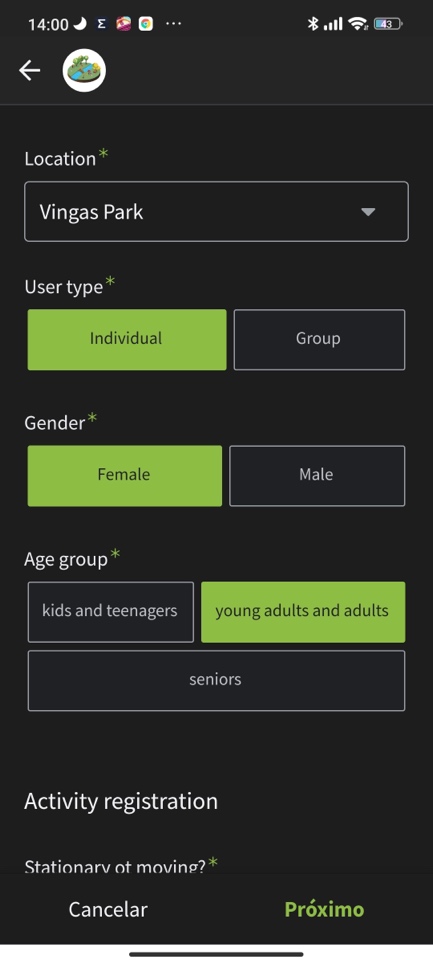 | 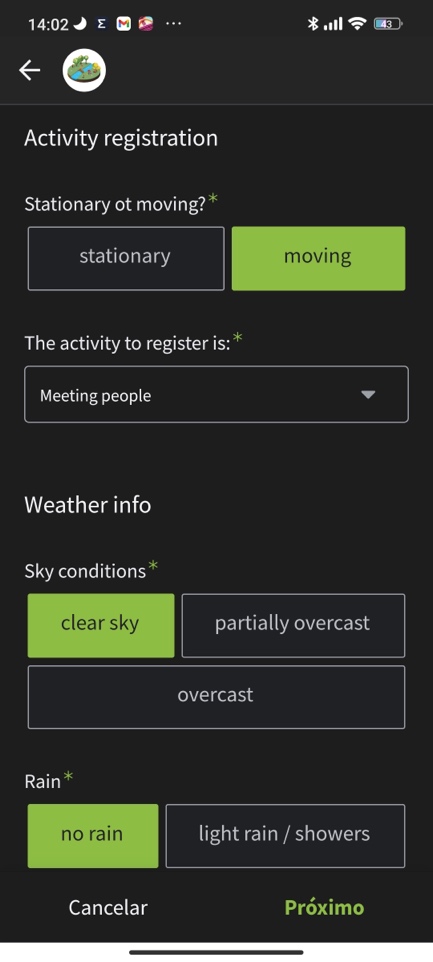 |
| d | e | f |

Figure S1 – App screenshots for: (a) and (b)results section with charts showing results for observations per park and users per activity from a field survey in Vilnius, Lithuania; (c) language selection; (d) and (e) ‘New observation’ form showing the application of the rules for data consistency and optimisation, with (d) showing the form after selecting a ‘group’ entry, and (e) section for activity registration after selecting an ‘individual’ entry; (f) ‘New observation form (continued): activity registration section, with list of options for activity dependent on motion option.


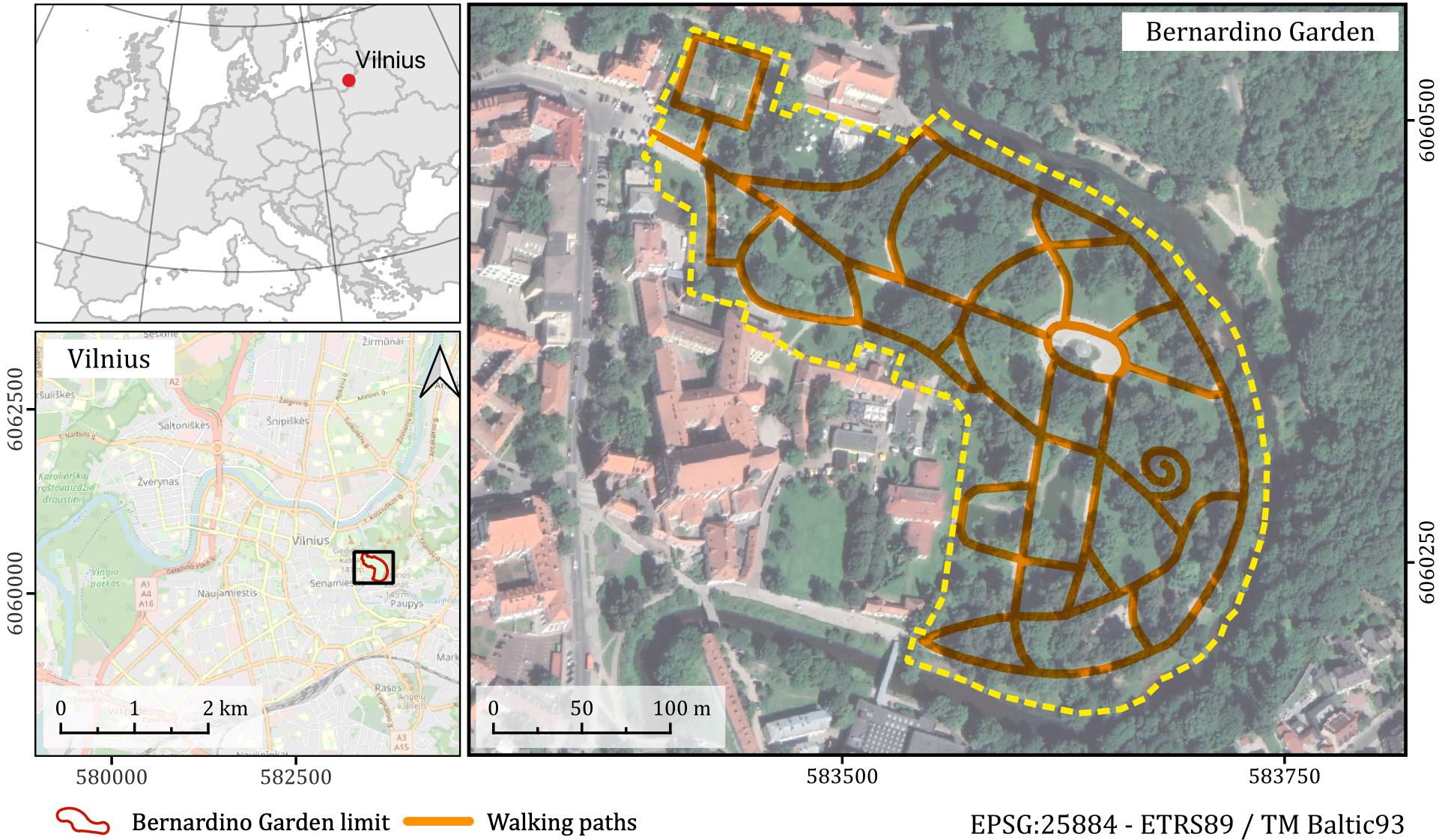


Figure S2 – Example of definition of walking paths to include in the field work, for a study in Vilnius, Lithuania.
